# Ventricular tachycardia with epicardial and pericardial fibrosis 6 months after resolution of subclinical COVID-19: a case report 

**DOI:** 10.1186/s13256-021-02782-w

**Published:** 2021-05-28

**Authors:** Jonathan Solaimanzadeh, Aaron Freilich, Michael R Sood

**Affiliations:** 1 Mount Sinai South Nassau, Oceanside, NY 11571 USA; 2 Icahn School of Medicine at Mount Sinai, New York, USA; 3 Mount Sinai Hospital, 148 Madison Ave, New York, NY 10029 USA

**Keywords:** SARS-CoV-2, COVID-19, Cardiovascular magnetic resonance, Late gadolinium enhancement, Ventricular tachycardia

## Abstract

**Background:**

Coronavirus disease 2019 (COVID-19) has been shown to have extensive effects on the cardiovascular system. Its long-term cardiac manifestations, however, remain unclear.

**Case presentation:**

We report the case of a Caucasian patient with a mild and self-limited presentation of COVID-19, with subsequent development, months later, of exertional dyspnea and non-sustained ventricular tachycardia, long after resolution of his illness and after returning to aerobic exercise. The patient had normal screening tests including electrocardiogram (ECG) and echocardiogram 4 months after his illness. Cardiac magnetic resonance imaging demonstrated epicardial and pericardial fibrosis of the right ventricle free wall and outflow tract and the pericardium over the anterior wall, 6 months following the initial infection. First abnormal ECG was recorded at month 7 following illness.

**Conclusions:**

This case suggests an insidious and possible long-term cardiac involvement and reflects the challenges in traditional workups and screening modalities in identifying cardiac involvement in COVID-19.

## Background

Coronavirus disease 2019 (COVID-19) has been shown to have a wide variety of clinical manifestations. It typically presents with a spectrum of symptoms ranging from no symptoms to an array of mild symptoms such as cough, headache, fever, or anosmia. Less common is a rapid progression of pneumonia, hypoxemia, and acute respiratory distress syndrome and/or death. Recent emerging evidence suggests a significant impact on the cardiovascular system. Proposed mechanisms involve direct viral- or inflammatory-mediated myocardial damage, endothelial dysfunction or plaque instability, arterial or venous thrombosis, and indirect hypoxic cell injury [1–11]. However, the long-term cardiac manifestations remain unclear.

Cardiovascular magnetic resonance imaging (CMR) is an advanced cardiac imaging modality that can directly visualize the cardiovascular system in any dimensional plane. It has excellent spatial and temporal resolution and is used to characterize myocardial tissues. It is considered the gold standard for assessing myocardial function. Clinically, it is utilized to aid in the assessment of various cardiomyopathies, myocarditis, and/or pericardial disease [12, 13]. Furthermore, gadolinium-based contrast agents used to demonstrate late gadolinium enhancement (LGE) have been shown to be an important prognosticator in various disease processes [14–17].

The following is a case report of a mild and self-limited presentation of COVID-19 with the subsequent development of cardiac symptoms months after its clinical resolution and CMR demonstrating epicardial and pericardial fibrosis.

## Case presentation

A 46-year-old athletic and otherwise healthy Caucasian male with a past medical history of mild hyperlipidemia was diagnosed with COVID-19 on March 17, 2020. He had symptoms of malaise, dry cough, anosmia, and a low-grade fever for 2 days prior to his test. His symptoms were self-limited and fully abated on day 5 after onset. He did not require supportive oxygen or hospitalization. Due to his self-limited illness, there were no laboratory data acquired such as cardiac biomarkers. He did not warrant nor was he treated with any pharmacotherapies or antibiotics. His baseline electrocardiogram (ECG) was reported normal including the QT interval. He resumed normal activity after resolution of his illness that month including vigorous aerobic exercise for the following 2–3 months and felt very close to his normal baseline functional status.

**Fig.1:**
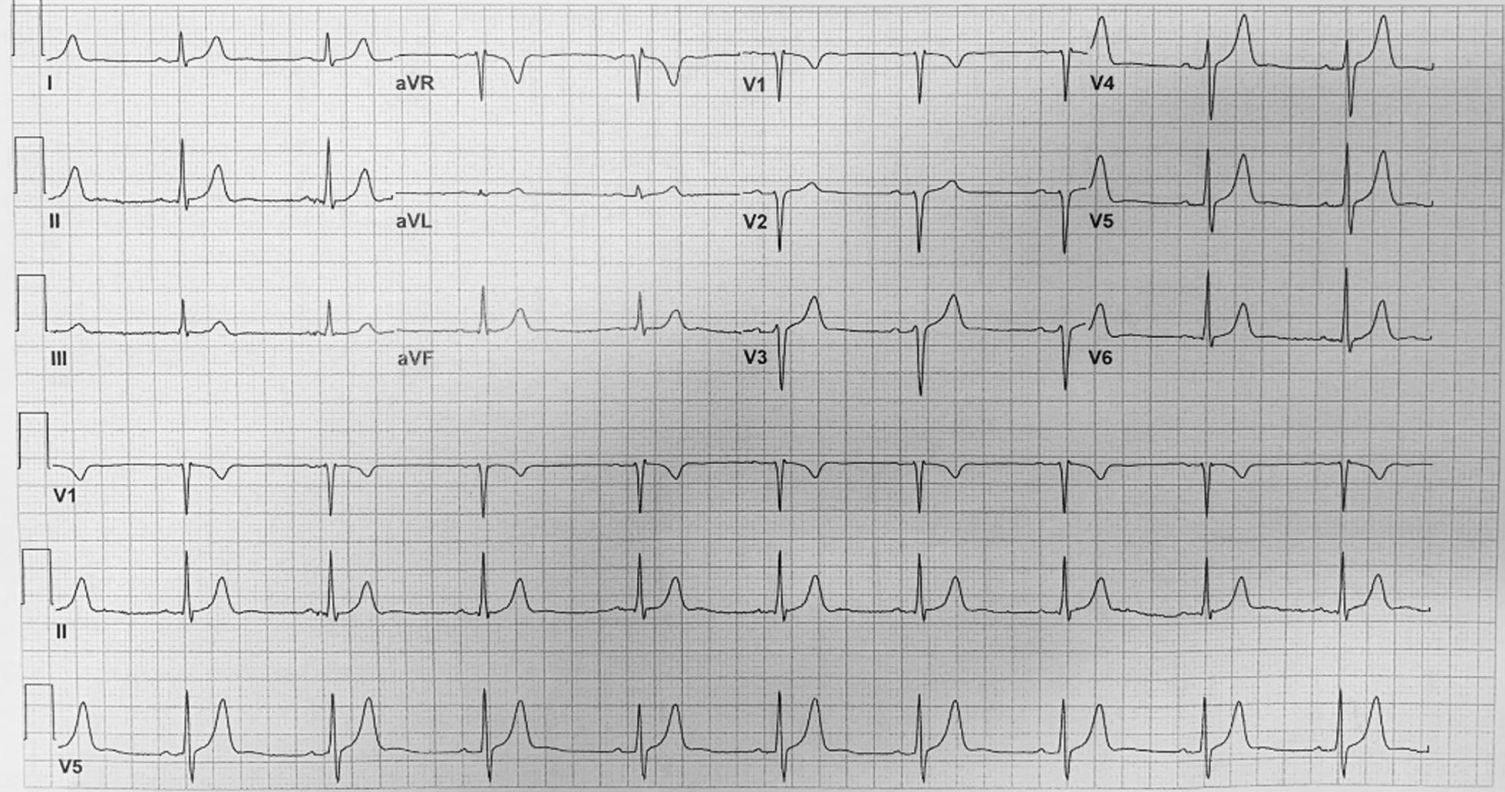
Electrocardiogram (ECG) 1, August 6, 2020, performed 5 months after illness reveals normal sinus rhythm. Prior ECGs were all normal as well

**Fig. 2:**
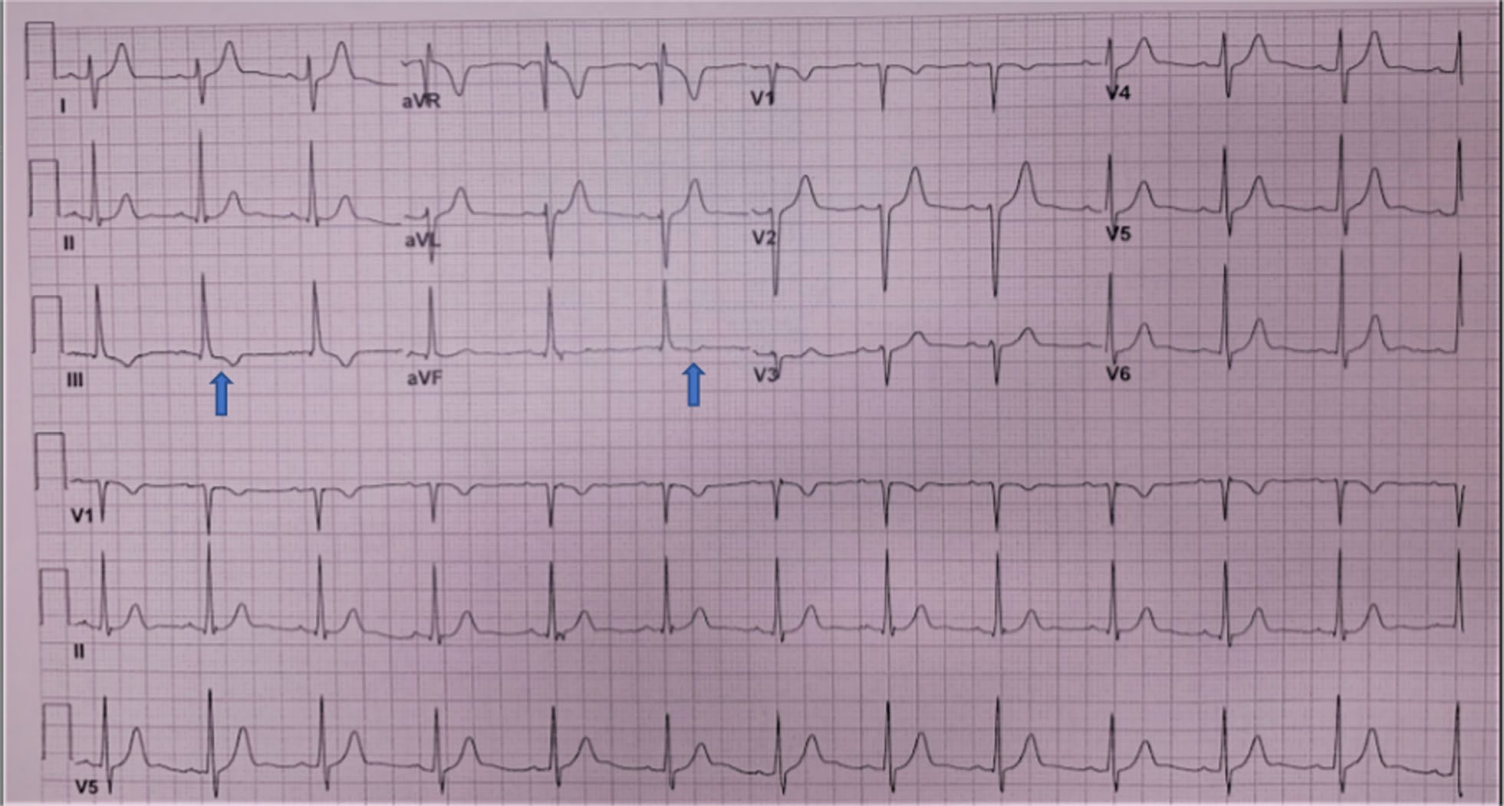
Electrocardiogram, September 8, 2020, reveals sinus rhythm with nonspecific ST changes inferiorly (arrows) and a rightward axis

Approximately 3 months after resolution of illness (June 2020), he began having frequent palpitations and exertional dyspnea. He attributed his symptoms to anxiety and some deconditioning from prior quarantine. He had COVID-19 antibodies checked at that time, which showed high titers. On month 4 after his diagnosis (July 2020), he had a chest x-ray, electrocardiogram, and echocardiogram which were all unremarkable. He underwent continuous Holter monitoring which showed frequent premature ventricular beats and multiple brief paroxysms of non-sustained ventricular tachycardia. His baseline ECG remained normal, as shown (Fig. 1). In September of the same year (month 7 following illness), his repeat ECG showed subtle abnormalities (Fig. 2).

**Fig. 3:**
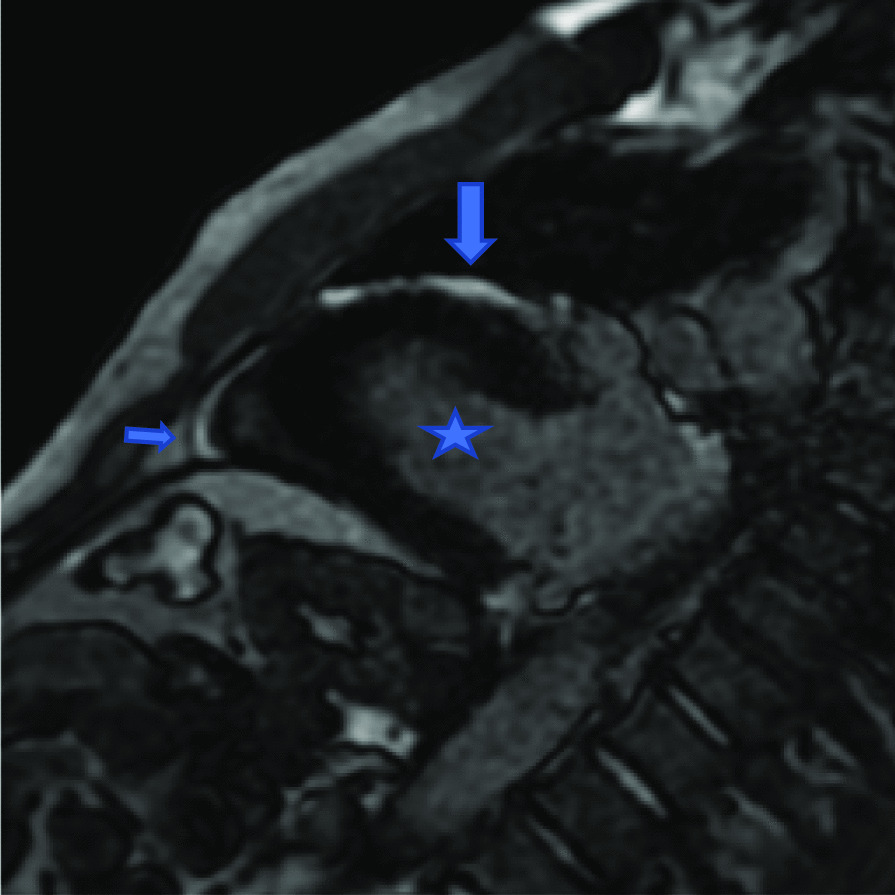
Cardiovascular magnetic resonance imaging, fast gradient echo, two-chamber view showing late gadolinium enhancement of the pericardium over the left ventricular anterior wall (arrow) and a trivial pericardial effusion (small arrow). Star denotes left ventricular cavity. Star denotes the left ventricle

**Fig. 4:**
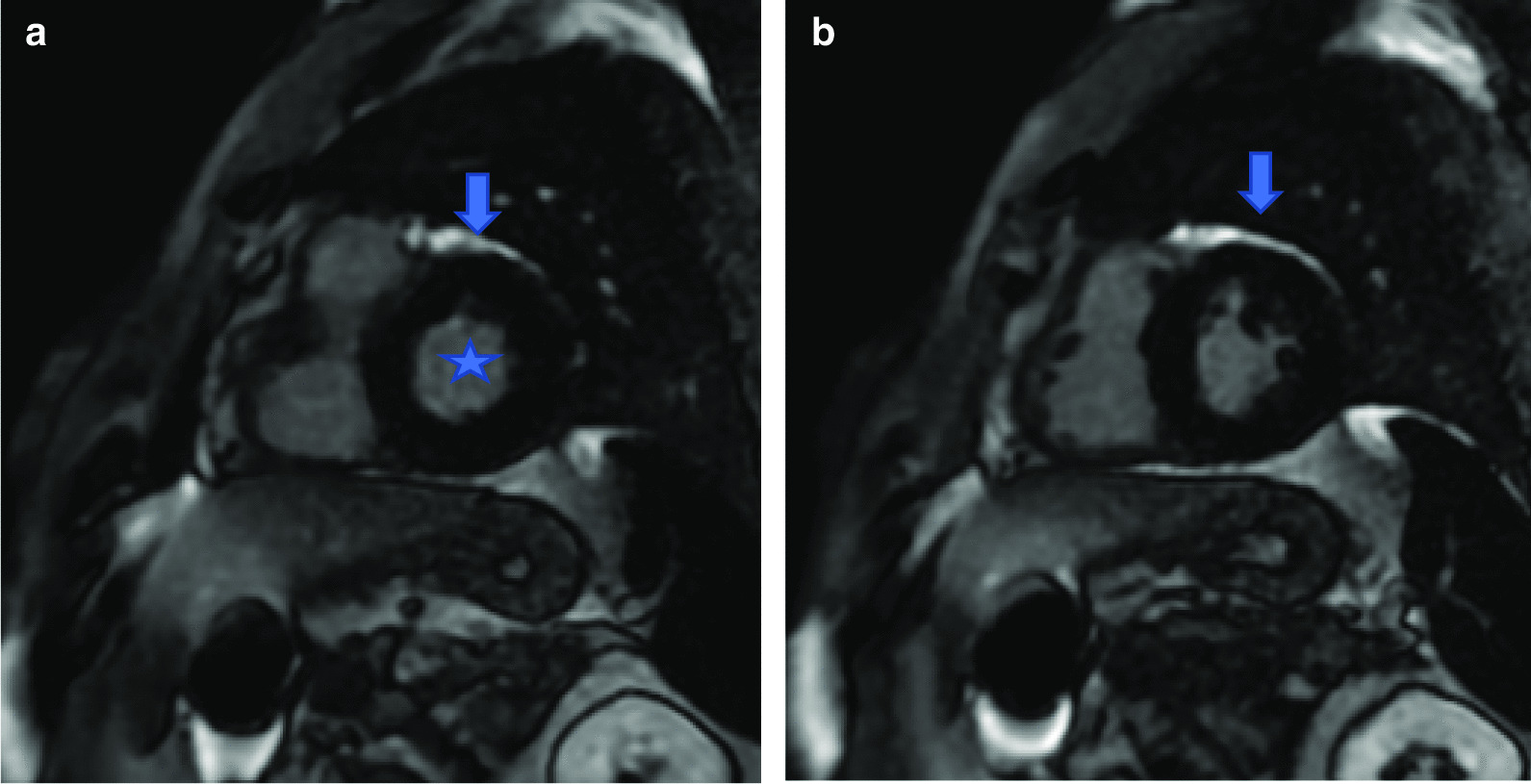
Cardiovascular magnetic resonance imaging, fast gradient echo, short-axis view from basal segment (**a**) to mid segment (**b**) showing late gadolinium enhancement of the pericardium over the anterior and anteroseptal walls (arrows). Star denotes left ventricular cavity. Star denotes the left ventricle

**Fig. 5:**
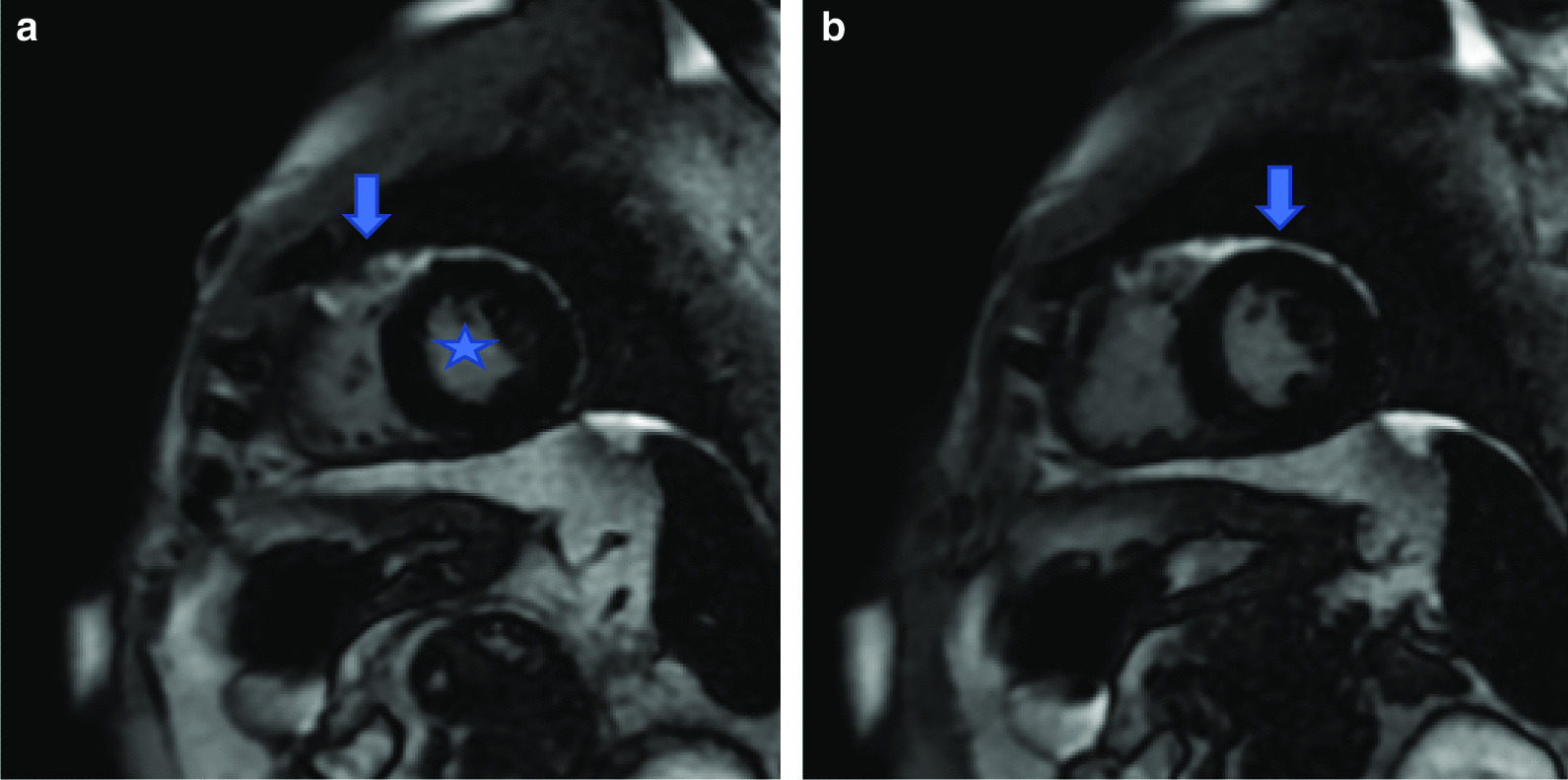
Cardiovascular magnetic resonance imaging, fast gradient echo, short-axis view of the mid to apical segment showing late gadolinium enhancement of the free wall of the right ventricle (**a**) and pericardium over the anterior wall, lateral wall (**b**) (arrows). Star denotes left ventricular cavity. Star denotes the left ventricle

At 5 months and 28 days after his illness, he underwent CMR using the General Electric Signa Artist 1.5 Tesla with field of view 36 x 32 mm, slice thickness 8 mm, 0 mm spacing, matrix 200 x 200 pixels mm, number of excitations 1. Gadolinium-enhanced imaging was performed approximately 10 min after administration of 0.1 mmol/kg body weight of gadobutrol (Gadovist; Bayer). Findings showed normal left and right ventricular systolic function with mild left ventricular hypertrophy, with prominent LGE involving epicardial and pericardial fibrosis of the basal to apical anterior wall and at the basal to mid anterior septum and right ventricular free wall with a trivial pericardial effusion (Figs. 3, 4, 5).

## Discussion

Emerging evidence has shown myocardial involvement in patients with covid-19. In one prospective, observational cohort study, 100 patients who had COVID-19 underwent CMR which revealed cardiac involvement in 78 percent of patients with the average follow-up time of 71 days. More than half of the patients had ongoing myocardial inflammation, which was independent of preexisting conditions and their severity of illness [18]

In another study, 26 competitive athletes who were diagnosed with the COVID-19 infection, all of which were either asymptomatic or presented with mild symptoms (sore throat, shortness of breath, myalgias, fever underwent CMR. Only four subjects (15%) had CMR findings suggestive of myocarditis. Pericardial effusion was present in only 2 patients [19]. Lui Haung, *et al.*, demonstrated 58% of patients had abnormal CMR findings, 54% with myocardial edema and LGE in 31% of patients in a retrospective study with an average patient age of 38-years-of age and follow-up time from illness to scan of 47 days [20]. Despite emerging data, much remains uncertain about the true prevalence as well as the long-term cardiac effects in covid-19. Many of the patients studied have had their CMR performed within a short window of their covid-19 illness and usually due to evident overlapping cardiac symptoms. Our case shows one of the longer CMR scans done from time of illness documenting covid-19 involvement to date.

Our patient appears to have developed symptoms of palpitations and dyspnea 3 months after his clinical resolution of covid-19, which is markedly unusual in the timing of his presenting cardiac symptoms. It is unclear if he had underlying inflammation or subclinical myocarditis, however, his echocardiogram and ECG were both notably normal, even on month 4 after illness. He also had high covid-19 antibody titers at that time, which suggested immunity. Furthermore, his CMR (6 months after illness) showed LGE with the absence of T2 weighted myocardial edema, which may reflect less of an acute myocardial injury response and more so a chronic process of fibrosis or scar remodeling [21–24]. It is also unclear if his trivial pericardial effusion seen on CMR at the time of scan was representing a resolving effusion or a dynamic intra-cardiac process (his echocardiogram done 2 months earlier had no effusion). Similarly, his new ECG abnormalities noted on month 7 after illness also correlate in an unusually similar time presentation, as illustrated (Image 1-2).

Our patient may have also had insidious or subclinical cardiac involvement that may have been exacerbated by returning to heavy aerobic exercise once he felt recovered. This may explain the large time gap between his recovery of covid-19 and the development of cardiac symptoms and findings. There is emerging evidence that myocarditis in covid-19 can be detected in athletes via CMR despite a normal ECG, echocardiogram and/or other laboratory or imaging parameters [25]. This may also support a low threshold to pursue advanced imaging modalities such as CMR, if clinically indicated. Lastly, our patient did not have screening cardiac biomarkers, which have been shown as a good utility in a large systemic review by Shafi *et al.* for cardiac involvement during active covid-19 infection [26]. This was due to his self-limited illness and the need for quarantine, for which he did not seek or warrant medical care and may represent many recovered patients.

### Follow-up

The patient is to undergo further ambulatory telemetry monitoring. Pharmacotherapy with beta blockade has been initiated with improvement in his palpitations. Electrophysiological testing with electrical mapping may be considered on his course if persistent ventricular tachycardia is detected or based on the development of other high-risk clinical features, and it may help to correlate electrical or ectopic origins with LGE patterns. Cardiac ablation and/or an implantable defibrillator may also be indicated in the future depending on his clinical course, although not warranted at this time. Finally, a follow-up CMR may also be considered to reassess fibrosis patterns or further myocardial involvement.

## Conclusions

This case suggests an insidious and atypical presentation of the cardiac manifestations of COVID-19 and may suggest possible long-term cardiac involvement, with demonstrated LGE on CMR 6 months following illness and new ECG findings at month 7. Furthermore, it illustrates the challenges in traditional workups and screening modalities in identifying patients with potential cardiac involvement and highlights the clinical utility of CMR in such patients. Clinicians should have a high index of clinical suspicion regardless of normal screening tests such as ECG or even echocardiogram and should consider close follow-up or monitoring in patients returning to heavy exercise after COVID-19 recovery. Future long-term and larger studies are recommended to better understand the long-term cardiac manifestations in COVID-19.

## Data Availability

Not applicable.

## Abbreviations

COVID-19: Coronavirus disease 2019
CMR: Cardiovascular magnetic resonance
LGE: Late gadolinium enhancement
ECG: Electrocardiogram
